# Prevalence and Molecular Characterization of *Toxoplasma gondii* and *Toxocara cati* Among Stray and Household Cats and Cat Owners in Tehran, Iran

**DOI:** 10.3389/fvets.2022.927185

**Published:** 2022-06-22

**Authors:** Poorya Karimi, Soheila Shafaghi-Sisi, Ahmad Reza Meamar, Gelareh Nasiri, Elham Razmjou

**Affiliations:** ^1^Department of Parasitology and Mycology, School of Medicine, Iran University of Medical Sciences, Tehran, Iran; ^2^Department of Microbiology, School of Medicine, Shahid Beheshti University of Medical Sciences, Tehran, Iran

**Keywords:** *Toxoplasma gondii*, *Toxocara* spp., zoonosis, human, cat, public health, Tehran, Iran

## Abstract

*Toxoplasma gondii* and *Toxocara* spp. are the most critical parasites common between humans and cats. The close association of cats with humans in urban areas persuaded us to investigate the prevalence of these parasites in stray and household cats and their possible role in the owners' infection. Herein, 132 and 33 fecal samples of stray and household cats, respectively, and 33 blood samples of their owners were collected in Tehran, Iran. The prevalence of *T. gondii* was determined by targeting the *B1* gene in the feces of stray and household cats and the blood of cat owners. Furthermore, genotypes of *T. gondii* were identified based on the multilocus genotyping of BTUB, GRA6, SAG3, and APICO loci. *Toxocara* spp. were detected by targeting the second internal transcribed spacer (ITS-2) of the ribosomal DNA of these parasites in the cats' feces and the humans' blood. Also, *Toxocara* IgG was assessed in the human serum samples. The *B1* gene amplification showed that 15.2% of stray cats, 18.2% of household cats, and 51.5% of cat owners were infected with *T. gondii*. The multilocus sequence analysis revealed the predominance of genotype I of *T. gondii* in stray cats and genotype II of *T. gondii* in household cats and cat owners. The amplifying of ITS-2 revealed a high prevalence of *T. cati* infection (47.0%) in stray cats, whereas no infection was found in the feces of household cats or the serum of cat owners. Likewise, *Toxocara* IgG was not detected in the serum of humans. The lower prevalence of *T. gondii* in stray/household cats than in the cat owners indicates the limited impact of close contact with infected cats in human toxoplasmosis. However, the high prevalence of *T. cati* infection in stray cats can cause contamination of the environment by excreting eggs that may lead to infecting humans through soil or water. Therefore, public health education in urban management planning is necessary for routine urban cat deworming programs and for training the healthcare workers to prevent, control, and treat these infections.

## Introduction

Cats (*Felis catus*) have lived in human societies for thousands of years, with a population of over 600 million worldwide. They are the most popular companion animal globally. Approximately 373 million cats were kept as pets in the world in 2018. (https://www.statista.com/statistics/1044386/dog-and-cat-pet-population-worldwide/). However, it is difficult to determine the number of cats that live freely in human societies (1). Cats harbor some important zoonotic parasites that can affect public health. Toxoplasmosis and toxocariasis are the most important parasitic diseases common between humans and cats (2).

Toxoplasmosis, caused by *Toxoplasma gondii*, is one of the most common protozoan parasitic infections in humans and animals. It is estimated that about two billion of the world's population are chronically infected with this obligate intracellular protozoan parasite (3). Toxoplasmosis generally remains asymptomatic in people with effective immune responses. However, the acute disease may manifest symptoms such as fever, lymphocytosis, and lymphadenitis. The severe form of the disease rarely leads to encephalitis, pneumonia, muscle disorders, and death. Furthermore, early infection during pregnancy may cause abortion (3). The *T. gondii* infections are acquired by consuming undercooked meat containing tissue cysts or contaminated food and water with oocysts defecated from cats that sporulate in the soil during 1–5 days (4, 5).

The oocysts of *Toxoplasma, Neospora*, and *Hammondia* parasites are morphologically indistinguishable. Consequently, molecular methods apply to differentiate *Hammondia*-like organisms in the feline feces (6). Molecular analysis has categorized *T. gondii* into three genotypes I, II, and III. Genotype II is the most prevalent genotype in humans (7, 8), although animal toxoplasmosis is more closely related to genotypes II and III (9). Whereas genotype I of *T. gondii* is more implicated in acute and lethal infections in humans and animals (10).

Stray and household cats, as the definitive hosts of *T. gondii* in urban communities, through a close relationship with humans, seem to be the leading cause of human infection by passing oocysts in feces (5). Hence, information about the prevalence of *T. gondii* in stray and household cats and cat owners is necessary to identify the possible role of cats in infecting humans.

Toxocariasis, caused by zoonotic *Toxocara* species, is one of the most frequently reported parasitic zoonosis in humans worldwide (11). Members of the *Toxocara* genus are among the most common helminths in canids and felids. They include essential human and animal health species, such as *Toxocara cati, Toxocara canis*, and *Toxascaris leonina*. Epidemiological studies indicate 1–100% of *T. canis* in dogs and 3.2–91% of *T. cati* in cats (12). The high amount of adult nematodes in the intestines of the definitive hosts leads to the contamination of playgrounds and urban areas that increases the risks of toxocariasis in humans, especially in children. Humans are infected by accidental ingestion of embryonic eggs or by ingesting undercooked meat containing encysted larvae (12). Toxocariasis clinical manifestations are visceral larva migrans, ocular larva migrans, latent toxocariasis, and neurological toxocariasis (12). The close association of cats with humans in urban areas makes them an important source of *Toxocara* infection. Therefore, knowing the exact number of infected cats is very effective for making the necessary decisions to prevent and control this disease. Limited studies on the prevalence of *Toxocara* spp. in Iran (13–16) persuaded us to investigate the prevalence of these parasites in stray and household cats and their possible role in the owners' infection.

We applied the molecular analyses approach to fill the gap in our understanding of the prevalence of *T. gondii* and *Toxocara* spp. in stray and household cats and determine the possible role of household cats in the infection of their owners. To reach these purposes, we determined the prevalence of *T. gondii* and *Toxocara* spp. by molecular detection of the *B1* gene of *Toxoplasma* and the ITS-2 region of the ribosomal DNA of *Toxocara* spp. in the feces of stray and household cats and the blood of cat owners in Tehran, Iran. In addition, genotypes of *T. gondii* were determined to identify the possible role of cats in infecting humans based on the multilocus genotyping of BTUB, GRA6, SAG3, and APICO loci.

## Materials and Methods

### Study Areas

**Figure 1:**
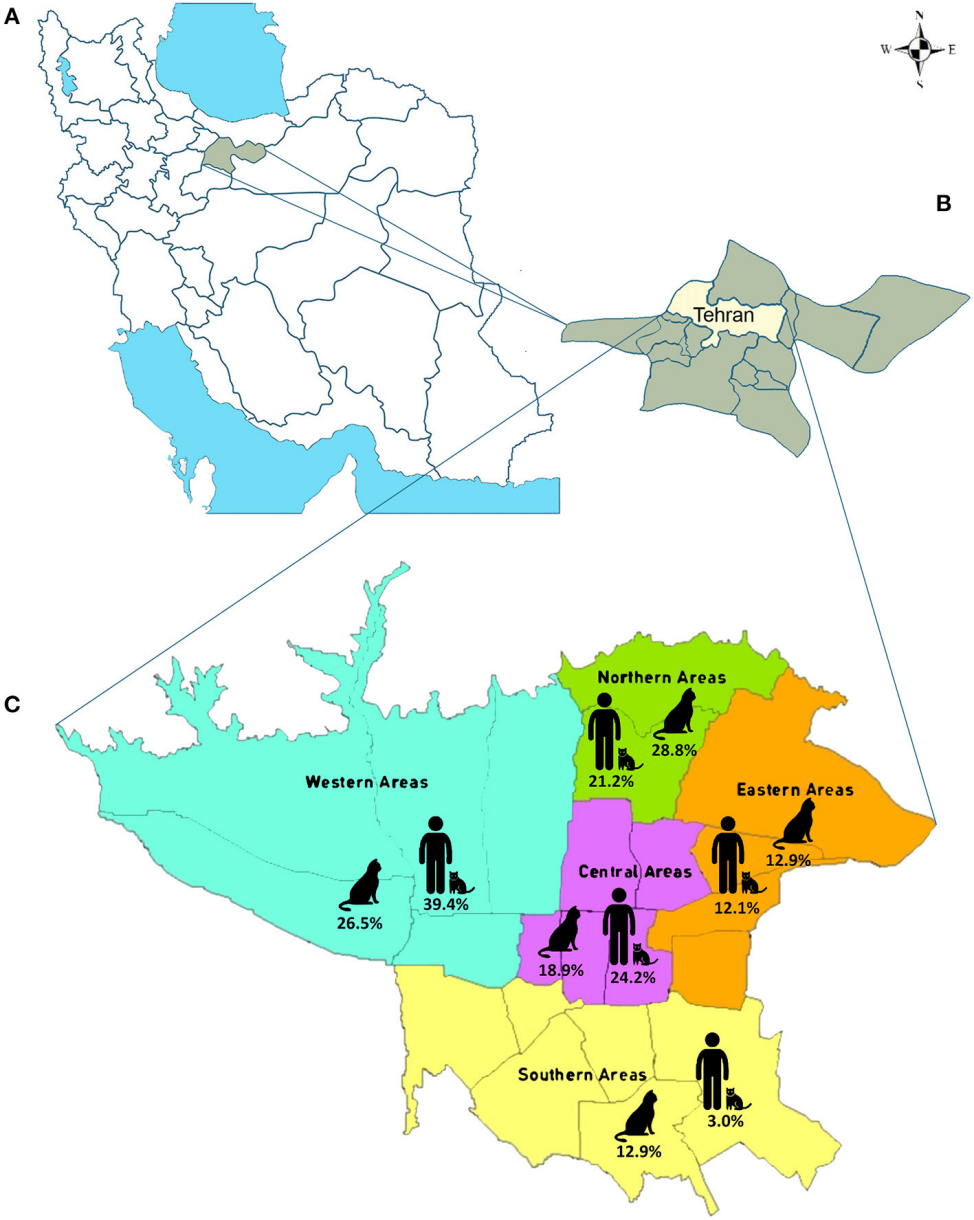
The map of the study area. **(A)** Map of Iran. Tehran Province is indicated by green. **(B)** Map of Tehran Province. Tehran is highlighted in yellow. **(C)** Map of Tehran. The five study areas are indicated, and the percentage of the stray and household cat fecal samples and blood of cat owners collected from each region are shown.

The study area was in Tehran (35.6892° N, 51.3890° E), the largest city and the capital of Iran (Figure 1). It covers an area of more than 730 km^2^ and has a population of 8.694 million. The climate of Tehran is generally mild and pleasant in spring and autumn, hot and dry in summer, and cold and wet in winter. The average annual temperature in Tehran is 16.4°C, and the mean annual rainfall is 220 mm.

### Questionnaires

The cat questionnaire was designed to include information on cat demographic data (sex, age, breed, weight, and residence area). There was no history of anti-parasitic treatments in stray cats, although they confidently did not take these drugs at the sampling time. The questionnaire of household cat owners collected information on demographic data (gender, age, occupation, residence area, and education). The cat owners declared that their cats had received anthelmintic treatments six months before sampling. The questionnaires are available in the Supplementary Material.

### Sample Collection

The stray cats injured in accidents or diseases have been transferred to veterinary clinics or pet hospitals in different parts of Tehran by animal-loving people or animal welfare associations. From 132 stray cats referred to these centers from different regions of Tehran's open public areas (north, south, east, west, and center), visibly-excreted fecal pellets were collected from October 2017 through March 2018. Thirty-three fecal samples were collected from household cats, and 33 blood samples were taken from their owners from January through September 2019. Six and seven milliliters of venous blood were collected in two tubes with and without EDTA to obtain the buffy coat and serum. Collected samples were transferred immediately to the research laboratory of the Department of Parasitology and Mycology, Iran University of Medical Sciences, for further analysis. Serum and buffy coats were stored at −80°C until further investigation. The *Toxocara* IgG in serum samples was evaluated with an ELISA commercial kit (*Toxocara* IgG ELISA kit; NovaTec Immunodiagnostic GmbH, Germany) following the manufacturer's instructions.

### Fecal Samples Preparation

Fecal samples of stray and household cats (*n* = 165) were submitted to the sucrose flotation procedure for concentrating cysts/oocysts of parasites (17). Six to eight grams of each sample was dissolved into 50 mL PBS until a homogeneous suspension was prepared. Then, the suspension was passed through three layers of gauze to eliminate large particles. The fecal suspension was centrifuged at 800×g for 5 min. For isolating cysts/oocysts, 50 mL PBS was added to the sediment. After complete mixing, 25 mL of suspension was layered gently over 20 mL of 1 M sucrose solution (specific gravity 1.13) in a 50 mL cleaned conical tube to form two completely distinct phases. The tubes were centrifuged at 800 × g for 5 min at 4°C. The interface and the upper layer of the sucrose were collected by a disposable pipette to a 15 mL clean conical tube. Then, the collected supernatant containing the purified cysts/oocysts of parasites was recentrifuged and washed three times with PBS to remove the residual sucrose. Finally, 100 mg of the sediment was re-suspended in 200 μL 2% polyvinylpolypyrrolidone (PVPP) in PBS, mixed thoroughly, and after keeping at −20°C for 24 h, stored at −80°C until submitted to DNA extraction procedures.

### DNA Extraction

The genomic DNA of parasites was extracted from fecal samples using the QIAamp DNA Mini Kit (QIAGEN Ltd., Hilden, Germany cat. no. 51306) and from serum or buffy coat using a QIAamp DNA Blood Minikit (QIAGEN Ltd., Hilden, Germany cat. no. 51104) according to the manufacturer's instructions. The isolated DNA was stored at −20°C until used in PCR reactions.

### PCR Amplification and Molecular Identification

Molecular identification of *T. gondii* was performed by a PCR assay based on the 194-bp fragment amplification of the *B1* gene to screen the *T. gondii* DNA in the cat fecal samples and the buffy coat of blood samples of household cat owners. The *B1* gene of *T. gondii* is a multi-copy sequence-specific and highly conserved gene in all *T. gondii* strains, making it highly useful in the molecular detection of the parasite (18). The PCR amplification was conducted by the primers designed by Schwab and McDevitt (19) and the PCR procedures introduced in our previous report (18). In addition, the multi-locus genotyping of *T. gondii* in the *B1*-positive samples was performed based on the BTUB, GRA6, SAG3, and APICO genetic markers (4, 20) by primers designated by Su et al. (4). The nested PCR was performed with Taq DNA Polymerase Master Mix (Amplicon III, Denmark, cat. no. 180301) in the conditions described by Arshadi et al. (18) (Table 1). The genotype of *T. gondii* was determined after sequencing the amplified fragment of each marker by internal amplification primers in both directions (Macrogen Inc., Seoul, South Korea).

**Table 1:** Primer sequences and PCR conditions were used for the molecular identification and characterization of *Toxoplasma gondii, Toxocara spp*., and *Toxascaris leonina* in the present study.

**Target species**	**Gene/ Marker**	**Primer nucleotide Sequences (5′-3′)**	**Amplicon size (bp)**	**Ref**.	**Cycling conditions**	**Ref**.
*T. gondii*	*B1*	F: GGAACTGCATCCGTTCATGAG R: TCTTTAAAGCGTTCGTGGTC	194	(19)	30 sec/95°C, 50 sec/55°C, 20 sec/72°C, 40 cycles	(18)
*T. gondii*	APICO	F: TGGTTTTAACCCTAGATTGTGG R: AAACGGAATTAATGAGATTTGAA F: TGCAAATTCTTGAATTCTCAGTT R: GGGATTCGAACCCTTGATA	639	(4)	PCR 1: 30 sec/95°C, 30 sec/50°C, 30 sec/72°C, 35 cycles PCR 2: 20 sec/95°C, 15 sec/50°C, 20 sec/72°C, 40 cycles	(18)
*T. gondii*	BTUB	F: TCCAAAATGAGAGAAATCGT R: AAATTGAAATGACGGAAGAA F: GAGGTCATCTCGGACGAACA R: TTGTAGGAACACCCGGACGC	411	(4)	PCR 1: 30 sec/95°C, 30 sec/50°C, 30 sec/72°C, 35 cycles PCR 2: 20 sec/95°C, 15 sec/48°C, 20 sec/72°C, 40 cycles	(18)
*T. gondii*	GRA6	F: ATTTGTGTTTCCGAGCAGGT R: GCACCTTCGCTTGTGGTT F: TTTCCGAGCAGGTGACCT R: TCGCCGAAGAGTTGACATAG	344	(4)	PCR 1: 30 sec/95°C, 30 sec/50°C, 30 sec/72°C, 35 cycles PCR 2: 20 sec/95°C, 15 sec/50°C, 20 sec/72°C, 40 cycles	(18)
*T. gondii*	SAG3	F: CAACTCTCACCATTCCACCC R: GCGCGTTGTTAGACAAGACA F: TCTTGTCGGGTGTTCACTCA R: CACAAGGAGACCGAGAAGGA	226	(4)	PCR 1: 30 sec/95°C, 30 sec/50°C, 30 sec/72°C, 35 cycles PCR 2: 20 sec/95°C, 15 sec/52°C, 20 sec/72°C, 40 cycles	(18)
*T. cati*	ITS-2	F: GGAGAAGTAAGATCGTGGCACGCGT R: TTAGTTTCTTTTCCTCCGCT	370	(21)	20 sec/94°C, 30 sec/58°C, 30 sec/72°C, 35 cycles	(21)
*T. canis*	ITS-2	F: AGTATGATGGGCGCGCCAAT R: TTAGTTTCTTTTCCTCCGCT	380		20 sec/94°C, 30 sec/58°C, 30 sec/72°C, 35 cycles	
*T. leonina*	ITS-2	F: CGAACGCTCATATAACGGCATACTC R: TTAGTTTCTTTTCCTCCGCT	300		20 sec/94°C, 30 sec/58°C, 30 sec/72°C, 35 cycles	

Molecular identification of *Toxocara* spp. and *T. leonina* was performed by species identification PCR procedures to target unique regions of the second internal transcribed spacer (ITS-2) of the ribosomal DNA of *T. canis, T. cati*, and *T. leonina*. The amplification of ITS-2 was performed according to the primers and PCR conditions specified by Jacobs et al. (21) in the cat fecal samples and the serum samples of household cat owners. The PCR product of 370 bp from *T. cati*, 380 bp from *T. canis*, and 300 bp from *T. leonina* were amplified by the Tcat1-NC2, Tcan1-NC2, and Tleo1-NC2 primer sets, respectively (Table 1).

PCR reactions were conducted on the samples using primer pairs and conditions listed in Table 1, along with the genomic DNA of the related parasite as a positive control and sterile nuclease-free water as a negative control. The PCR products were analyzed by electrophoresis on 1.5% (w/v) agarose gel.

### Sequencing and Phylogenetic Analyses

The PCR-positive amplification products were analyzed and excised from 1% (w/v) agarose gels after electrophoresis to purify using the MinElute gel extraction kit (QIAGEN Ltd., Hilden, Germany), directly sequenced in both directions using forward and reverse primers (Macrogen Inc., Seoul, South Korea). The obtained sequences were verified by the Chromas software (Technelysium Pty Ltd., Queensland, Australia). Then, each parasite sample's corresponding forward and reverse sequences were aligned and assembled using MEGA X software (22). Finally, the final sequences were blasted (http://blast.ncbi.nlm.nih.gov) to identify the genotype of *T. gondii*, confirm the PCR-positive samples of *T. cati*, and compare the homology of the obtained sequences of each parasite isolate with the corresponding sequences earlier deposited in the GenBank database.

**Figure 2:**
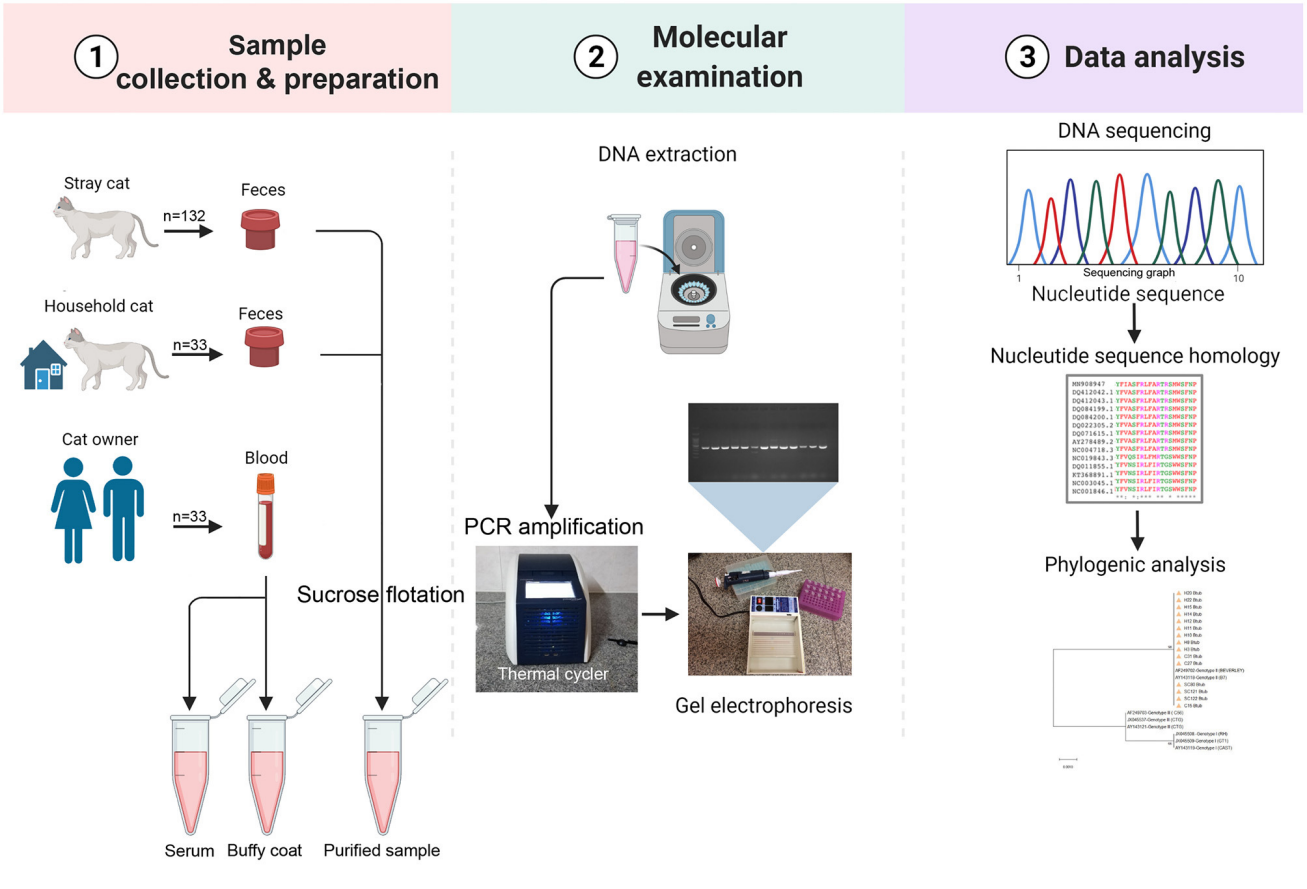
Sample analysis procedures. Image created in Biorender.com.

The obtained sequences were deposited in GenBank under accession numbers LC700048–LC700062, LC700063–LC700066, LC700067–LC700080, and LC700081–LC700088 for BTUB, GRA6, SAG3, and APICO loci of *T. gondii*, respectively, and LC700099–LC700103 for the ITS-2 of *T. cati*. The Phylogenetic trees were constructed by maximum likelihood (ML) algorithm with evolutionary distances calculated using the Kimura-2 parameter model and 1000 bootstrap values in the MEGA X software. Figure 2 represents a conceptual overview of sample analysis procedures.

### Statistical Analyses

The descriptive analysis evaluated the prevalence of parasites based on age, sex, breed, weight, and the urban region in cats and age, gender, occupation, residence area, and education in cat owners. Potential associations between qualitative variables were estimated by Chi-square tests with a 95% confidence interval (CIs) in SPSS 24.0 (SPSS Inc., Chicago, IL, USA) to reveal statistically significant differences (*P-*value <0.05).

## Results

### Study Population

The demographic characteristics of 165 cats enrolled in the study, 132 strays and 33 household cats, were categorized into two age groups under or over 1 year old, three breeding groups of DSH (Domestic Short Hair), DLH (Domestic Long Hair), and the Persian cat, three weight groups of <2 kg, between 2 and 4 kg and >4 kg, and five geographic locations of north, center, south, west, and east of Tehran (Table 2). Thirty-three cat owners participated in the investigation, 57.6% men and 42.4% women. The mean age was 38.1 ± 2.15 (M ± SEM) years, ranging from 25 to 68 years. The demographic data of participants included in this study are shown in Table 3.

**Table 2:** Demographic characteristics of the stray and household cats and the prevalence (%) and the number positive (N) of *Toxoplasma gondii and Toxocara cati* in Tehran.

**Factors**	**Stray cats**					**Household cats**		
	**Samples** **% (N)**	***Toxoplasma gondii***		***Toxocara cati***		**Samples** **% (N)**	***Toxoplasma gondii***	
		**% (N)**	***p***	**% (N)**	***p***		**% (N)**	***p***
**Age**								
<1 year	47.0% (62)	25.8% (16)	0.001	50.0% (31)	0.511	15.2% (5)	20.0% (1)	0.909
>1 year	53.0% (70)	5.7% (4)		44.3% (31)		84.8% (28)	17.9% (5)	
**Sex**								
Male	54.5% (72)	16.7% (12)	0.595	51.4% (37)	0.265	51.5% (17)	17.6% (3)	0.935
Female	45.5% (60)	13.4% (8)		41.7% (25)		48.5% (16)	18.7% (3)	
**Breed**								
DSH	72.7% (96)	14.6% (14)	0.761	50.0% (48)	0.275	33.3% (11)	27.2% (3)	0.509
DLH	25.8% (34)	17.7% (6)		41.2% (14)		9.1% (3)	0.0% (0)	
Persian	1.5% (2)	0.0% (0)		0.0% (0)		57.6% (19)	15.8% (3)	
**Weight**								
<2 kg	47.0% (62)	19.4% (12)	0.447	50.0% (31)	0.123	8.2% (6)	16.7% (1)	0.866
2–4	40.2% (53)	11.3% (6)		37.8% (20)		57.6% (19)	21.1% (4)	
>4 kg	12.8% (17)	11.8% (2)		64.7% (11)		24.2% (8)	37.5% (1)	
**Urban Region**								
North	28.8% (38)	15.8% (6)	0.761	47.4% (18)	0.733	21.2% (7)	28.6% (2)	0.180
Center	18.9% (25)	20.0% (5)		56.0% (14)		24.2% (8)	12.5% (1)	
South	12.9% (17)	11.8% (2)		41.2% (7)		3.0% (1)	100.0% (1)	
West	26.5% (35)	17.1% (6)		48.6% (17)		39.4% (13)	7.7% (1)	
East	12.9% (17)	5.9% (1)		35.3% (6)		12.1% (4)	25.0% (1)	
**Total**	100.0% (132)	15.2% (20)		47.0% (62)		100.0% (33)	18.2% (6)	

*DLH, Domestic long hair; DSH, Domestic short hair*.

**Table 3:** Demographic characteristics of the cat owners in Tehran and the prevalence (%) and the number positive (N) of *Toxoplasma gondii*.

**Factors**	**Samples % (N)**	***Toxoplasma gondii***	
		**% (N)**	***p***
**Age**			
20–29	27.3% (9)	33.3% (3)	0.047
30–39	33.3% (11)	36.4% (4)	
40–49	18.2% (6)	100.0% (6)	
>50	21.2% (7)	57.1% (4)	
**Gender**			
Male	57.6% (19)	36.8% (7)	0.049
Female	42.4% (14)	71.4% (10)	
**Urban Region**			
North	21.2% (7)	57.1% (4)	0.792
Center	24.2% (8)	62.5% (5)	
South	3.0% (1)	0.0% (0)	
West	39.4% (13)	46.1% (6)	
East	12.1% (4)	50.0% (2)	
**Occupation**			
Self-employment	36.3% (12)	50.0% (6)	0.521
Employee	36.3% (12)	41.7% (5)	
Housekeeper	27.2% (9)	66.7% (6)	
**Education**			
Diploma	27.2% (9)	33.3% (3)	0.393
Undergraduate	39.4% (13)	53.8% (7)	
Postgraduate	33.3% (11)	63.6% (7)	
**Total**	100.0% (33)	51.5% (17)	

### Prevalence of *Toxoplasma* and *Toxocara* Among Stray and Household Cats and Their Owners

Monitoring of the *T. gondii B1* gene showed that 15.2% (20/132; 95% CI 10.0–22.2) of stray cats, 18.2% (6/33; 95% CI 8.6–34.4) of household cats, and 51.5% (17/33; 95% CI 35.2–67.5) of cat owners were infected with *T. gondii*. There were no statistical differences between the prevalence of *T. gondii* in stray and household cats. However, the prevalence of *T. gondii* in cat owners was statistically higher than in both groups of cats. The prevalence of *T. gondii* infection relative to demographic variables of stray cats, household cats, and cat owners are shown in Tables 2, 3. There was no statistically significant relationship between sex, breed, weight, urban region, and infection with *T. gondii* in the stray and household cats. Moreover, statistical analysis found no association between urban region, occupation, and education of cat owners and *T. gondii* infection. However, age affected the prevalence of *T. gondii* in stray cats and cat owners. The highest prevalence of *T. gondii* infection was detected in stray cats under 1 year old (25.8%; 95% CI 10.0–22.2; *p* = 0.001) and cat owners aged 40–49 years (100.0%; 95% CI 61.0–100.0; *p* = 0.047). Furthermore, the prevalence of *T. gondii* in the women cat owners 71.4% (10/14; 95% CI 45.4–88.3) was higher than in the men 36.9% (7/19; 95% CI 19.1–59.0) (*p* = 0.049).

The species identification PCRs of *Toxocara* spp. and *T. leonina* revealed a high prevalence of *T. cati* infection (62/132; 47.0%; 95% CI 38.7–55.4) and no *T. canis* or *T. leonina* in stray cats. The ribosomal DNA of *T. canis, T. cati*, and *T. leonina* was not detected in the feces of household cats or the serum of cat owners (0/33; 0.0%; 95% CI 0.0–10.4). In addition, the ELISA showed 33 sera samples of cat owners to be *Toxocara* IgG negative. Consequently, the *T. cati* infections in stray cats were significantly higher than in household cats and cat owners. Table 2 shows the prevalence of *T. cati* infection relative to demographic variables of stray cats. There was no statistically significant relationship between any demographic variables and infection with *T. cati* in the stray cats.

Overall, molecular examination showed at least one *T. gondii* or *T. cati* infection occurred in 73 of 132 (55.3%; 95% CI: 47.7–63.6%) stray cats. In addition, coinfections with both parasites were observed in 9 of 132 stray cats (6.8%; 95% CI: 3.0–11.4%). However, coinfections did not ensue in household cats or their owners.

### *Toxoplasma gondii* Multilocus Genotyping of Isolates From Stray Cats, Household Cats, and Cat Owners

The multi-locus genotyping of 43 *B1* positive isolates of *T. gondii* were performed based on the BTUB, GRA6, SAG3, and APICO genetic markers. The genotype of 26 (60.5%; 95% CI 45.6–73.6) *B1* positive isolates were determined with at least one locus, 12, 4, and 10, from stray cats, household cats, and cat owners, respectively. The BTUB, GRA6, SAG3, and APICO loci were amplified and successfully sequenced in 15 (34.9%), 4 (9.3%), 14 (32.6%), and 8 (18.6%) of the *B1* positive isolates, respectively (Table 4). The sequencing analysis of four markers showed 9 (34.6%) genotype I, 12 (46.2%) genotype II, and one (3.8%) genotype III (Table 4). Four (14.5%) isolates were inconsistently identified as the genotypes I or II with two markers (Table 4; Supplementary Table S1).

**Table 4:** Multi-locus genotyping of *Toxoplasma gondii* isolates from *B1* positive samples of stray cats (SC), household cats (C), and cat owners (H).

**Sample code**	**Genotype/genetic marker**				
	**BTUB**	**GRA6**	**SAG3**	**APICO**	**Final**
SC8	na	II	II	II	II
SC26	na	III	III	III	III
SC48	na	na	I	I	I
SC54	na	na	I	na	I
SC77	na	na	I	na	I
SC80	II	na	na	na	II
SC102	na	na	I	I	I
SC109	na	na	I	I	I
SC112	na	na	I	na	I
SC117	na	na	I	na	I
SC121	II	na	na	II	II
SC122	II	na	I	I	I, II
C15	II	na	I	na	I, II
C21	na	na	I	na	I
C27	II	na	na	na	II
C31	II	na	na	na	II
H3	II	na	I	I	I, II
H8	II	na	na	na	II
H9	na	na	I	na	I
H10	II	na	na	na	II
H11	II	na	na	na	II
H12	II	I	na	na	I, II
H14	II	na	na	na	II
H15	II	II	na	na	II
H20	II	na	na	na	II
H22	II	na	na	na	II

*na, not successfully amplified*.

**Figure 3:**
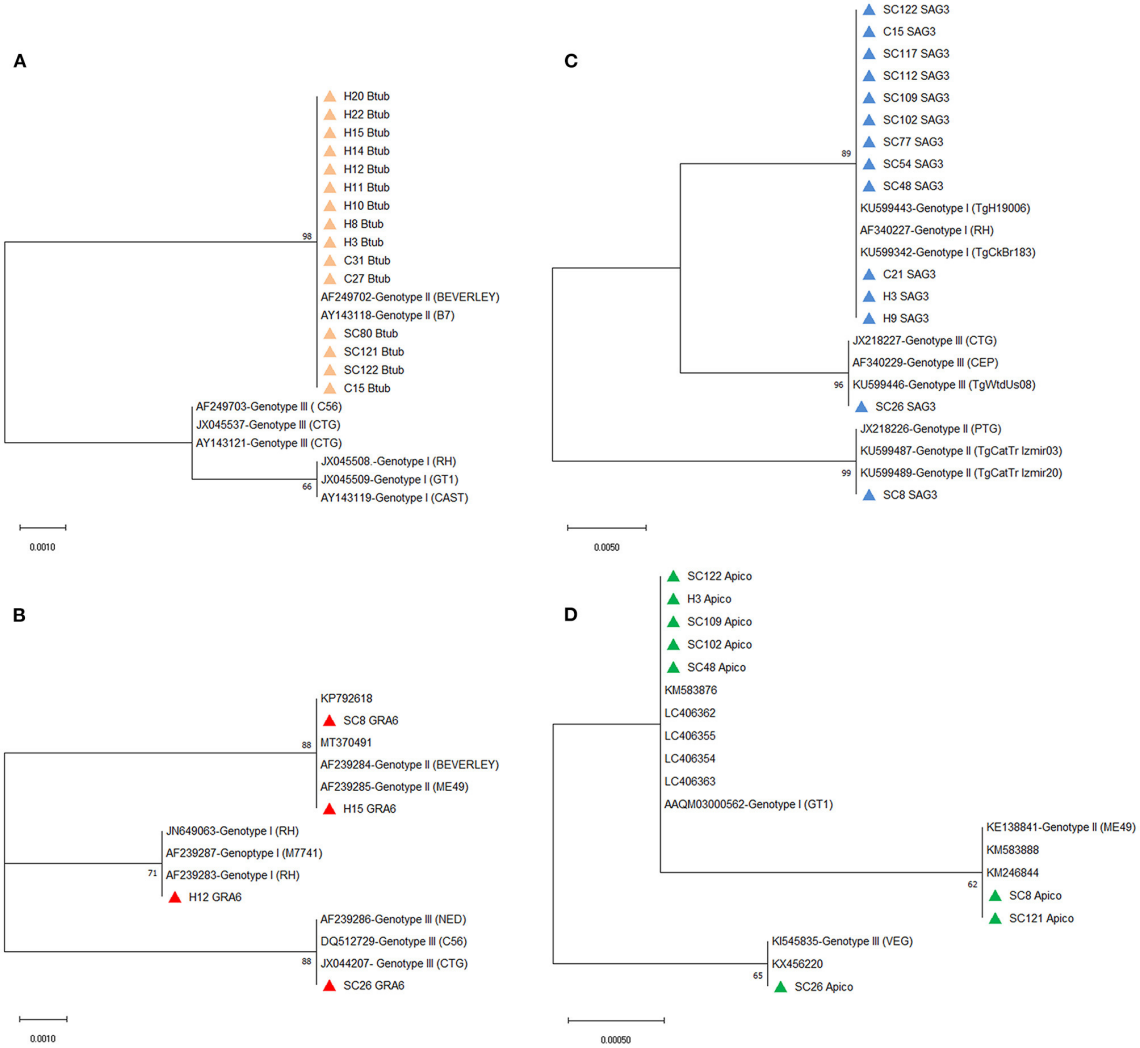
Phylograms of *Toxoplasma gondii* genotypes were inferred based on the nucleotide sequences of the **(A)** BTUB, **(B)** GRA6, **(C)** SAG3, and **(D)** APICO. The evolutionary relationship of *T. gondii* genotypes was constructed by the Maximum Likelihood method and Kimura 2-parameter model, based on the nucleotide sequences of **(A)** BTUB, **(B)** GRA6, **(C)** SAG3, and **(D)** APICO genetic markers of *T. gondii* isolated from stray cats [SC], household cats [C], and cat owners [H] retrieved from this study (colored triangles) compared with reference sequences of *T. gondii* genotype I, II, and III from GenBank. Bootstrap values obtained from 1,000 replicates are indicated on branches in percentage; only bootstrap values >50% are displayed. Evolutionary analyses were conducted in MEGA X.

The multiple alignments of BTUB gene sequences identified the 15 amplified isolates as genotype II from stray cats (three isolates), household cats (three isolates), and cat owners (nine isolates). In addition, sequencing analysis revealed 100% homology between 15 BTUB genotype II isolates and the reference sequence of AF249702 (BEVERLEY) and AY143118 (B7) for genotype II (Supplementary Table S1). Only one isolate from the household cat (C15) and the corresponding owner (H15) were amplified and successfully sequenced at the BTUB locus. Phylogenetic analysis of the BTUB gene located all 15 BTUB amplified isolates in one cluster with genotype II sequence references (AF249702 and AY143118), with a 98% bootstrap value (Figure 3A). Sequencing analysis revealed that four GRA6 amplified isolates belonged to genotype I (one cat owner isolate), II (one stray cat and one cat owner isolate), and III (one stray cat isolate). Moreover, the nucleotide sequence alignment and phylogenetic analysis of GRA6 isolates with the corresponding retrieved genotype I, II, and III sequences of GenBank showed 100% homology (Supplementary Table S1), with 71% or 88% bootstrap value (Figure 3B). Further sequence alignment and phylogenetic analysis of the SAG3 locus identified genotypes I (8 isolates), II (one isolate), and III (one isolate) in stray cats. In contrast, only genotype I was verified in household cats (two isolates) and cat owners (two isolates). Figure 3C illustrates the identified isolates of the SAG3 locus classified in three clusters with sequence references representing genotypes I, II, and III with 89%, 99%, and 96% bootstrap support, respectively. The nucleotide sequence alignment revealed eight APICO amplified isolates belonged to genotype I (four stray cat isolates and one cat owner isolate), II (two stray cat isolates), and III (one stray cat isolate) with 100% homology with sequence references representing genotype I, II and III (Supplementary Table S1). The phylogenetic analysis of APICO isolates was clustered with the corresponding retrieved genotype I, II, and III sequences of GenBank with high bootstrap values (Figure 3D).

The multilocus sequence analysis of 26 *Toxoplasma*-positive isolates revealed genotype I of *T. gondii* was predominant in stray cats (7/12; 58.3%; 95% CI 32.0–80.7), although genotype II of *T. gondii* was predominant in household cats (2/4; 50.0%; 95% CI 15.0–85.0) and cat owners (7/10; 70.0%; 95% CI 39.7–89.2). In addition to genotype I (58.3%), genotype II (3/12; 16.7%; 95% CI 4.7–44.8), genotype III (1/12; 8.3%; 95% CI 1.5–35.4), and the mix of genotypes I and II (1/12; 8.3%; 95% CI 1.5–35.4) were identified in stray cats. Genotype I (1/4; 25.0%; 95% CI 4.6–69.9) (1/10; 10.0%; 95% CI 1.8–40.4) and the mix of genotypes I and II (1/4; 25.0%; 95% CI 4.6–69.9) (2/10; 20.0%; 95% CI 5.7–51.0) showed less distribution in household cats and cat owners than genotype II (Table 4).

### The Nucleotide Sequence Analysis of the ITS-2 Region Confirmed the Predominance of *T. cati* in Stray Cats

**Figure 4:**
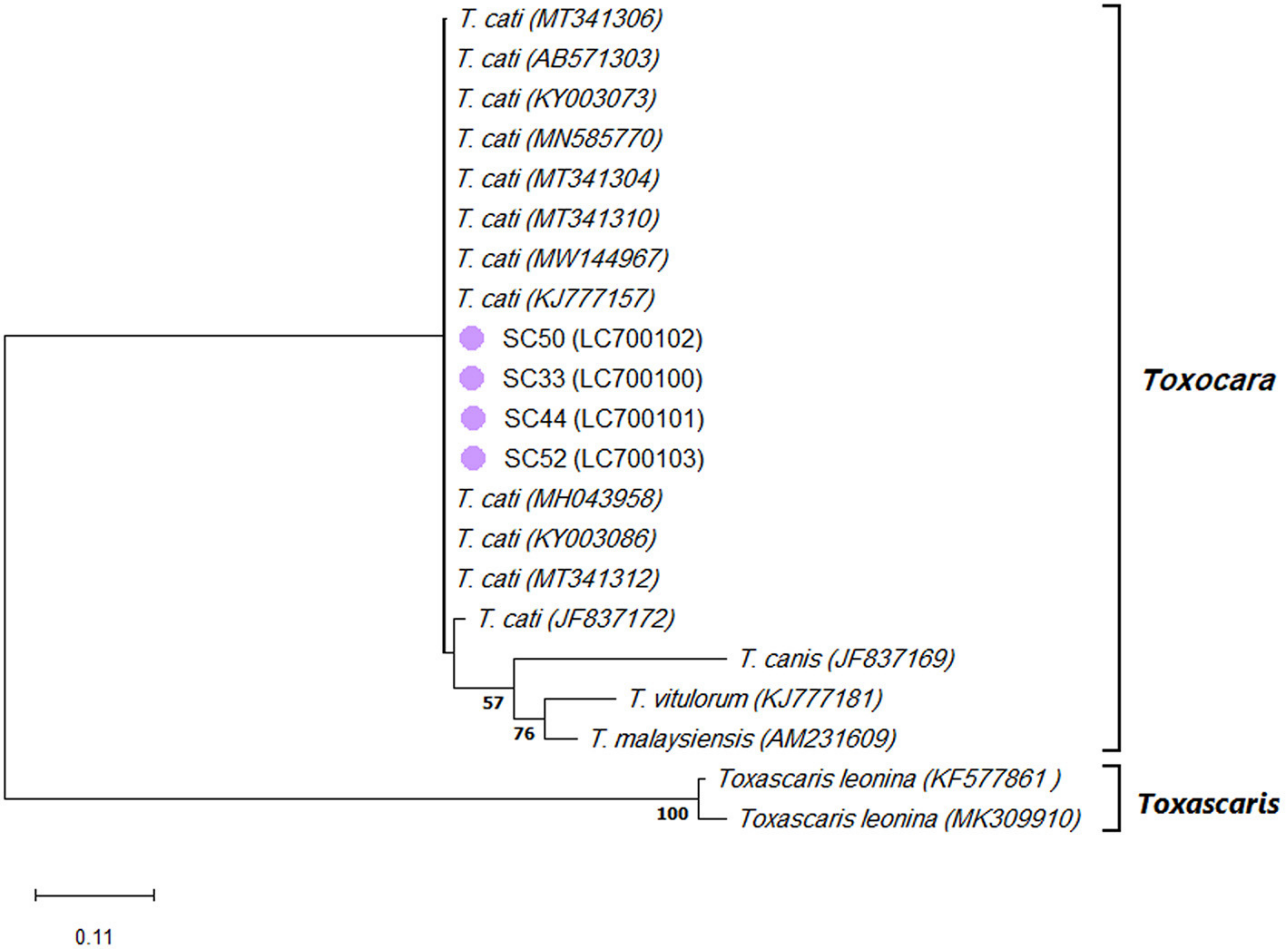
Phylogram of *Toxocara* spp. and *Toxascaris leonina* was inferred based on the nucleotide sequences of the ITS-2 region of the ribosomal DNA. The evolutionary relationship of *Toxocara* spp. *and T. leonina* was constructed by the Maximum Likelihood method and Kimura 2-parameter model, based on the nucleotide sequences of the ITS-2 region of the ribosomal DNA of *Toxocara cati* isolated from stray cats [SC] retrieved from this study (purple circles) compared with nucleotide sequences of *Toxocara* spp. and *T. leonina* from GenBank. Bootstrap values obtained from 1000 replicates are indicated on branches in percentage; only bootstrap values >50% are displayed. Evolutionary analyses were conducted in MEGA X.

The species identification PCR procedures of the ITS-2 region of the ribosomal DNA of *T. canis, T. cati*, and *T. leonina* showed 62 positive samples of *T. cati* in stray cats. Sequencing a 370-bp amplified fragment of four *T. cati* PCR-positive samples confirmed the results. In addition, the nucleotide sequencing analysis of four *T. cati* isolates revealed 100% homology with the corresponding fragment of the published ITS-2 sequences of *T. cati* retrieved from GenBank (MT341311 and KJ777157). Further phylogenetic analysis revealed four *T. cati* isolates in one cluster with the published ITS-2 sequences of *T. cati* retrieved from GenBank, with a 94% bootstrap value (Figure 4).

## Discussion

Using the molecular analysis methods, we detected *T. gondii* and *Toxocara* spp. infections in the feces of stray and household cats and the blood of cat owners by amplifying the *B1* gene of *T. gondii* and the ITS-2 region of the ribosomal DNA of *Toxocara* spp. Furthermore, by employing the multilocus genotyping of *T. gondii* based on BTUB, GRA6, SAG3, and APICO loci, we identified genotyping and genetic variability of *T. gondii* isolated from cats and humans in Tehran, a metropolitan area in Iran. We found a higher prevalence of *T. gondii* in cat owners than in cats, affected by the age and gender of humans and only the age of stray cats. Although we identified a high prevalence of *T. cati* infection in stray cats, we did not detect *Toxocara* spp. or *T. leonina* in household cats or their owners. We also revealed the predominance of genotype I of *T. gondii* in stray cats and genotype II of *T. gondii* in household cats and cat owners.

Although infected cats usually shed the oocysts once in their lives and for a short time (23, 24), infected humans carry bradyzoites of parasite for long-lasting periods inside their cells, and latent infection may persist. These biological behaviors of *T. gondii* can explain the higher prevalence of DNA parasites in the cat owner's buffy coat than in cats' feces. The reported prevalence of *T. gondii* in cats in different parts of the world is variable according to the diagnostic methods used and the variety in geographic, environmental, and diet of studied cats. For example, the prevalence of *T. gondii* in stray cats in Tehran (15.2%), compared to other molecular studies in Iran, was higher than the previous prevalence reported in Mashhad (4.5%) (25) and Ahvaz (7.2%)(26), and lower than the prevalence (24.1%) reported in Shiraz (27). Furthermore, compared to other molecular studies in the world, it is higher than the previous prevalence reported in South Korea (4.7% and 1.3%) (28, 29), the USA (2%) (30), and Malaysia (13%)(31), and lower than the prevalence reported in South Korea (17.5% and 30.6%)(32, 33) and Pakistan (34%)(34). In addition, the prevalence of *T. gondii* infection in household cats in the current study (18.2%) was higher than the prevalence reported in Switzerland (0.58%) (35), Poland (2.4%) (36), Malaysia (4%) (31), Thailand (4.7%) (37) and Kenya (7.8%) (38), and lower than reported from Italy (20.5%) (39). To the best of our knowledge, this is the first study performed on the molecular prevalence of *T. gondii* infection in cat owners worldwide. A systematic review and meta-analysis estimated that the seroprevalence of *T. gondii* infection in the general Iranian population was 39.3%, reported at 49% in Tehran (40), which is very close to our results (51.5%). Our previous study (18) confirms that detecting DNA circulating in the blood is comparable to the seropositive results.

The investigation of possible factors associated with *T. gondii* infection showed it was more frequent in stray cats under 1 year old (*p* = 0.001), similar to a study conducted in South Korea (28). However, Khodaverdi et al. (25) in Mashhad and Kwak et al. (29) in South Korea had not observed any statistically significant relationship between the age of cats and the prevalence of *T. gondii* infection. Moreover, a significant difference between cat owners' age and the prevalence rate was observed, comparable to the highest incidence previously reported in the age group over 40 years (40) and the correlation between increasing age and the prevalence of infection (41). It is assumed that through passing the years, exposure to microorganisms increases, whether through contaminated food and water, contact with infected cats, or other risk factors. The higher *T. gondii* infection in the women cat owners observed in our study was not confirmed by the earlier seroprevalence studies (40).

Multi-locus genotyping of BTUB, GRA6, SAG3, and APICO loci of *Toxoplasma*-positive isolates revealed genotype I in stray cats and genotype II in household cats and cats' owners were predominant. In addition, three genotypes of *T. gondii* and the mix of genotypes I and II were typed in the stray cats of Tehran, which demonstrated more variables than previous studies. For example, genotype I (one case), III (32 cases), and the mix of I and III (two cases) in Ahvaz, southwest of Iran, by PCR-RFLP of SAG2 locus (26), or genotype II in Mashhad (one case), northeastern Iran, by PCR-RFLP of SAG3 locus (25), and in South Korea (two isolates) (28) by sequencing *SAG5D* and *SAG5E* genes were reported. Therefore, compared to our study, genotyping the limited number of samples or typing with just one locus in the mentioned reports is the foremost possible cause for not detecting all genotypes. Furthermore, as reported before, conflicting findings in multilocus typing could be seen regarding the small sample size (42).

Despite the ubiquity of *T. gondii* infections, the presence of comprehensive multi-locus studies investigating the *T. gondii* genetic diversity in household cats and their cat owners are lacking. However, most studies in Iran have reported the *T. gondii* genotype of different hosts or soil isolates based on the analysis of one or two loci (43–50). In limited studies performed on household cats, in Switzerland, mixed infections with types I and II in the only positive sample, and in Germany, type II (83.8%) and type III (1.4%) have been reported in household cats (35, 51). We characterized the simultaneous *T. gondii* infection in only a household cat (C15) and its owner (H15). The mix-infection of genotypes I and II in the cat and the genotype II in the cat owner was typed. Although we cannot definitively and accurately explain this finding, it is assumed that the cat was infected due to hunting or contact with contaminated soil through its roaming behavior, according to the declaration of the cat owner. Therefore, it is also possible that the cat and its owner were infected from two different sources.

The species identification PCRs of *Toxocara* spp. and *T. leonina* revealed a high prevalence of *T. cati* infection (62/132; 47.0%; 95% CI 38.7–55.4) and no *T. canis* and *T. leonina* in stray cats. The ribosomal DNA of *T. canis, T. cati*, and *T. leonina* was not detected in the feces of household cats or the serum of cat owners. The high prevalence of *T. cati* infection (47.0%) was identified in stray cats by detecting the ribosomal DNA of *T. cati* in the feces. This finding increased the concern about the potential role of the cats as significant reservoirs for humans, especially children. Nevertheless, we did not find any cases of infection in the feces of household cats or the serum of cat owners. However, the reported prevalence of *T. cati* infection in cats has a variety of ranges depending on geographical regions and the detection methods. A relatively limited number of studies in different parts of Iran and the world have investigated the molecular prevalence of these nematodes in the feces of stray cats. The prevalence of *T. cati* in the current study was higher than in other studies performed on stray cats in the different cities of Iran (16, 52, 53) and Malaysia (54). In this study, we performed PCR to detect the DNA of these nematodes in the serum of cat owners and the feces of household cats for the first time, which was confirmed by not detecting the *Toxocara* IgG in the sera of cat owners. However, the anti-*Toxocara* antibodies were reported in the sera of residents of northeastern Iran (7.2%) (55) and northern Iran (23.5%) (56). None of the household cats or their owners were positive, which could be due to the lack of risk factors such as younger age and living in rural areas (56) or the result of attention to hygiene principles, routine anthelmintic therapy, and adequate veterinary care. Nevertheless, the high prevalence of *T. cati* infection in stray cats can cause contamination of the environment by excreting eggs that lead to infecting humans through soil or water. Therefore, the control and treatment of infection in cats are necessary to reduce the source of human disease and other paratenic hosts.

The main limitation of this study was collected samples from the sick or injured stray cats brought to veterinary centers and pet hospitals. Therefore, the results might not be a good representation of the entire stray cat population in the area, and this limitation should be considered in further studies. Another limitation was the relatively small sample size of household cats and their owners compared to the stray cats, which might be affected the prevalence and risk factors of infections. Nevertheless, finding many cat owners who didn't have other pets or had no close contact with other animals and were willing to cooperate in the study was difficult. Therefore, future studies with larger sample sizes are recommended to confirm the reported results in this study.

## Conclusion

The high prevalence of *T. cati* infection (47%) in stray cats leads to considering the role of cats in human toxocariasis. Whereas the low prevalence of *T. gondii* in stray cats (15.2%) and household cats (18.2%) compared to their owners (51.5%) suggests a more subordinate role of direct contact with infected cats than consuming contaminated undercooked meat containing tissue cysts or contaminated food and water with sporulated oocysts in cases of human infections. The relatively high prevalence of *Toxocara* infection in cats necessitates attention to routine urban cat deworming programs and public health education in urban management to generally promote physical and mental health in the community and specifically live happily and healthy coexistence with these urban animals.

## Data Availability Statement

The datasets presented in this study can be found in online repositories. The names of the repository/repositories and accession number(s) can be found in the article/Supplementary Material.

## Ethics Statement

The studies involving human participants and animal study were reviewed and approved by the Iranian Ministry of Health, Treatment, and Medical Education. The experimental protocols were reviewed by the Ethics Committee of Iran University of Medical Sciences (Approval No IR.IUMS.FMD.REC 1396.31834). The patients/participants provided their written informed consent to participate in this study.

## Author Contributions

PK: conceptualization, methodology, validation, formal analysis, investigation, resources, data curation, and original draft writing. SS-S methodology, validation, formal analysis, investigation, resources, and data curation. ARM: validation, review, and editing of the final manuscript. GN: methodology and formal analysis. ER: conceptualization, methodology, validation, formal analysis, resources, data curation, original draft writing, review and editing of the final manuscript, visualization, supervision, project administration, and funding acquisition. All authors contributed to the article and approved the submitted version.

## Funding

The Iran University of Medical Sciences financially supported this study under grant number 96-03-30-31834 to ER.

## Conflict of Interest

The authors declare that the research was conducted in the absence of any commercial or financial relationships that could be construed as a potential conflict of interest.

## Publisher's Note

All claims expressed in this article are solely those of the authors and do not necessarily represent those of their affiliated organizations, or those of the publisher, the editors and the reviewers. Any product that may be evaluated in this article, or claim that may be made by its manufacturer, is not guaranteed or endorsed by the publisher.

## Abbreviations

APICO: apicoplast genetic marker
BTUB: Beta-tubulin
DLH: Domestic long hair
DSH: Domestic short hair
GRA6: granule dense 6
ITS-2: Internal transcribed spacer region 2
MEGA: molecular evolutionary genetic analysis
Kimura: 2-parameter
ML: maximum likelihood
NJ: neighbor-joining
nested PCR: nested polymerase chain reaction
PVPP: Polyvinylpolypyrrolidone
SAG3: Surface antigen 3
SNPs: single nucleotide polymorphisms.
