# Supplementary material for: Prevalence and Molecular Characterization of Toxoplasma gondii and Toxocara cati Among Stray and Household Cats and Cat Owners in Tehran, Iran
Source: Front Vet Sci. 2022 Jun 22;9:927185. doi: 10.3389/fvets.2022.927185 (PMC9257223; doi:10.3389/fvets.2022.927185)
Supplement: Supplementary file 1 [file Table_1.docx]

**TABLE S1** The multiple alignments nucleotide sequences of BTUB, GRA6, SAG3, and APICO locus from *Toxoplasma gondii* isolated in this study with reference sequences retrieved from GenBank, represent the position of intra-genotypic substitutions in Genotype I, II, and III of‎ *T. gondii.*

| **Isolate/access no** | **Nucleotide position** | | | | | | | | | |
| --- | --- | --- | --- | --- | --- | --- | --- | --- | --- | --- |
| **BTUB** | **34** | **99** | **136** | **139** | **299** |  |  |  |  |  |
| AF249702-Genotype II (BEVERLEY) | G | G | G | C | C |  |  |  |  |  |
| AY143118-Genotype II (B7) | . | . | . | . | . |  |  |  |  |  |
| AF249703-Genotype III ( C56) | C | C | C | G | . |  |  |  |  |  |
| AY143121-Genotype III (CTG) | C | C | C | G | . |  |  |  |  |  |
| JX045508.-Genotype I (RH) | C | C | C | G | T |  |  |  |  |  |
| AY143119-Genotype I (CAST) | C | C | C | G | T |  |  |  |  |  |
| SC80, SC121, SC122, C15, C27, C31, H3, H8, H10, H11, H12, H14, H15, H20, H22_BTUB | . | . | . | . | . |  |  |  |  |  |
|  |  |  |  |  |  |  |  |  |  |  |
| **GRA6** | **113** | **143** | **178** | **234** | **243** |  |  |  |  |  |
| JN649063-Genotype I (RH) | C | G | C | G | A |  |  |  |  |  |
| AF239283-Genotype I (RH) | . | . | . | . | . |  |  |  |  |  |
| AF239285-Genotype II (ME49) | T | T | . | . | G |  |  |  |  |  |
| AF239284-Genotype II (BEVERLEY) | T | T | . | . | G |  |  |  |  |  |
| AF239286-Genotype III (NED) | . | T | T | A | . |  |  |  |  |  |
| DQ512729-Genotype III (C56) | . | T | T | A | . |  |  |  |  |  |
| SC8_GRA6, H15_GRA6 | T | T | . | . | G |  |  |  |  |  |
| SC26_GRA6 | . | T | T | A | . |  |  |  |  |  |
| H12_GRA6 | . | . | . | . | . |  |  |  |  |  |
|  |  |  |  |  |  |  |  |  |  |  |
| **SAG3** | **44** | **64** | **68** | **100** | **107** | **109** | **116** | **124** | **139** |  |
| AF340227-Genotype I (RH) | C | G | A | T | G | G | A | G | A |  |
| KU599342-Genotype I (TgCkBr183) | . | . | . | . | . | . | . | . | . |  |
| JX218226-Genotype II (PTG) | T | A | G | . | A | . | C | C | G |  |
| KU599487-Genotype II (TgCatTr Izmir03) | T | A | G | . | A | . | C | C | G |  |
| JX218227-Genotype III (CTG) | . | . | . | C | . | A | C | C | . |  |
| AF340229-Genotype III (CEP) | . | . | . | C | . | A | C | C | . |  |
| SC8_SAG3 | T | A | G | . | A | . | C | C | G |  |
| SC26_SAG3 | . | . | . | C | . | A | C | C | . |  |
| SC48, SC54, SC77, SC102, SC109, SC112, SC117, SC122, C15, C21, H3, H9_SAG3 | . | . | . | . | . | . | . | . | . |  |
|  |  |  |  |  |  |  |  |  |  |  |
| **APICO** | **156** | **533** |  |  |  |  |  |  |  |  |
| AAQM03000562-Genotype I (GT1) | T | C |  |  |  |  |  |  |  |  |
| KE138841-Genotype II (ME49) | C | . |  |  |  |  |  |  |  |  |
| KI545835-Genotype III (VEG) | . | T |  |  |  |  |  |  |  |  |
| SC8_APICO, SC121_APICO | C | . |  |  |  |  |  |  |  |  |
| SC26_APICO | . | T |  |  |  |  |  |  |  |  |
| SC48, SC102, SC109, SC122, H3_APICO | . | . |  |  |  |  |  |  |  |  |
|  |  |  |  |  |  |  |  |  |  |  |
