# Supplementary material for: Prevalence and Molecular Characterization of Toxoplasma gondii and Toxocara cati Among Stray and Household Cats and Cat Owners in Tehran, Iran
Source: Front Vet Sci. 2022 Jun 22;9:927185. doi: 10.3389/fvets.2022.927185 (PMC9257223; doi:10.3389/fvets.2022.927185)
Supplement: Supplementary file 2 [file Data_Sheet_1.PDF]

IRAN UNIVERSITY OF MEDICAL SCIENCES

Questionnaire form of household cat owners

Date: **Sample code:**

A) General Information

1- Age (year):…….

2- Gender: Male □ Female □

3- Occupation:……….

4- Residence Area:…………

6- Education:………….

B) Results

1- Result of *B1* gene PCR:

2- Result of BTUB gene nested PCR:

3- Result of GRA6 gene nested PCR:

4- Result of SAG3 gene nested PCR:

5- Result of APICO gene nested PCR:

6- Result of ITS-2 gene PCR:

7- Result of *Toxocara* IgG-ELISA: Negative □ Positive □

IRAN UNIVERSITY OF MEDICAL SCIENCES

Questionnaire form of stray/household cats

Date: **Sample code:**

A) General Information

1- Age (year):……. <1 year ☐ >1 year ☐

2- Sex: Male ☐ Female ☐

3- Breed: …… DSH ☐ DLH ☐ Persian ☐

4- Weight:…… <2kg ☐ 2-4kg ☐ >4kg ☐

5- Residence Area:……..

6- Date of receiving anthelmintic treatment:

B) Results

1- Result of *B1* gene PCR:

2- Result of BTUB gene nested PCR:

3- Result of GRA6 gene nested PCR:

4- Result of SAG3 gene nested PCR:

5- Result of APICO gene nested PCR:

6- Result of ITS-2 gene PCR:
